# Actively soniferous tropical reef fishes are diverse, vulnerable, and valuable

**DOI:** 10.1111/jfb.16030

**Published:** 2024-12-16

**Authors:** Emma Jayne Hodson, Kieran Cox, Francis Juanes, Audrey Looby

**Affiliations:** ^1^ Department of Biology University of Victoria Victoria British Columbia Canada; ^2^ College of Life and Environmental Sciences University of Exeter, Penryn Campus Penryn UK; ^3^ Department of Biological Sciences Simon Fraser University Burnaby British Columbia Canada; ^4^ Fisheries and Aquatic Sciences, Institute of Food and Agricultural Sciences University of Florida Gainesville Florida USA; ^5^ Nature Coast Biological Station, Institute of Food and Agricultural Sciences University of Florida Cedar Key Florida USA

**Keywords:** bioacoustics, ecoacoustics, marine, passive acoustic monitoring, sound production, soundscape

## Abstract

Active (i.e., intentional) fish sound production provides informative cues for numerous ecological functions, including larval recruitment or reproduction, and can facilitate monitoring and restoration. It is therefore important to have a holistic picture of actively soniferous tropical reef fish diversity, particularly in the face of growing threats such as noise pollution and habitat degradation. This study integrates fish biodiversity and sonifery datasets to assess the prevalence and ecological characteristics of actively soniferous tropical reef fishes. There are 258 known sound‐producing species, which span 46 families, encompass a variety of life‐history (e.g., lifespan) and distribution (e.g., depth) attributes, and include many vulnerable and commercially valuable species. Furthermore, up to 75% of tropical reef fish species are considered likely to produce active sounds. This synthesis should encourage a greater appreciation for active fish sound production in tropical reef environments and advance efforts to incorporate soundscape ecology into management and restoration strategies.

## 1 INTRODUCTION

Soundscapes—encompassing sounds from biotic, abiotic, and anthropogenic sources—are inextricably linked to tropical reef functioning (Lamont et al., 2022; Lindseth & Lobel, 2018). Many reef fish species produce active (i.e., intentional) sounds for communication in disturbance, aggression, and reproduction behaviors (Looby et al., 2022; Parmentier et al., 2021; Tricas & Boyle, 2014). These and other biological sounds provide navigational and settlement cues for fishes and invertebrates (Aoki et al., 2024; Simpson et al., 2008). Fish sounds also offer practical management applications, including ecological monitoring with passive acoustics (e.g., Desiderà et al., 2019; Lamont et al., 2022; Malinowski et al., 2019) or restoration with acoustic enrichment (e.g., Aoki et al., 2024; Gordon et al., 2019). The various uses of fish sounds can be disrupted, however, by the many anthropogenic threats facing reef environments, including noise pollution, habitat degradation, and ocean acidification (Duarte et al., 2021; Gordon et al., 2018, 2019; Munday et al., 2009).

Research on actively soniferous tropical reef fishes has grown dramatically in recent years, with resultant expectations that their ecological contributions are widespread (Lindseth & Lobel, 2018; Looby et al., 2022; Parmentier et al., 2021; Tricas & Boyle, 2014). Despite over a century of contemporary scientific research into fish sound production, until recently the sounds of coral reef fishes were rarely studied (Myrberg & Fuiman, 2002) and the extent of soniferous fishes globally remained unclear (Looby et al., 2022). Great strides have been made to counteract these knowledge gaps, including through species auditioning (e.g., Fetterplace et al., 2022; Parmentier et al., 2011), field observations (e.g., Tricas & Boyle, 2014), reviews of select taxa or regions (e.g., Colleye & Parmentier, 2012; Parmentier et al., 2021), and the creation of inventories of fish sound production knowledge (Looby et al., 2022, 2024). Tropical coral reefs are now among the most common habitats sampled in marine soundscape studies (Havlik et al., 2022) and acoustic monitoring is increasingly being incorporated into marine management strategies (e.g., McKenna et al., 2021). Awareness of fish sound production has been further advanced globally, for example through numerous calls for comprehensive repositories of underwater sound recordings such as a global library of underwater biological sounds (GLUBS; Parsons et al., 2022) or the Sea Sounds Portal (KAUST, 2024), though these aspirations have not yet fully come to fruition. Comprehensive sound production surveys and estimates based on taxonomy suggest that many reef fishes produce active sounds, at least within certain regions (Fish & Mowbray, 1970; Parmentier et al., 2021; Tricas & Boyle, 2014). A quantitative global synthesis of known reef fish sound‐producers and their traits would therefore add to these efforts and facilitate a broader understanding of fish sound contributions to underwater soundscapes, reef processes, and conservation.

To elucidate the prevalence and importance of actively soniferous (hereafter, soniferous) fish species to tropical reef environments globally, we integrated datasets of fish biodiversity and sonifery to describe their taxonomic, life‐history, distribution, vulnerability, and commercial attributes. We focused primarily on fish species that have been shown to produce active sounds auditorily or morphophysiologically but also explored the extent of tropical reef fishes considered likely to produce sound. While we do not delve into detailed descriptions of these taxa and their sound production here, we encourage those interested to explore other articles devoted to those purposes (e.g., Colleye & Parmentier, 2012; Parmentier et al., 2011).

## 2 METHODS

We collated data from three sources: FishSounds, a database of fish sound production information and recordings (Looby et al., 2021, 2022); FishBase, a global fish biodiversity database (Froese & Pauly, 2023); and the World Register of Marine Species (WoRMS), a global database of marine and other taxa that includes an ecological trait of confirmed or likely sonifery created through a collaboration between a GLUBS working group and FishSounds (Looby et al., 2024; WoRMS Editorial Board, 2024). We retrieved a list of soniferous fish species globally that had been studied in the scientific literature between 1874 and 2021 from the FishSounds Borealis data repository (Looby et al., 2021). We updated the species names to those accepted in the May 2023 version of FishBase (Froese & Pauly, 2023) and filtered for those that were listed as reef‐associated and native or endemic to saltwater, tropical environments, as categorized by the FishBase R package, rfishbase version 5.0.0 (Boettiger et al., 2012). We then extracted, explored, and visualized available taxonomic and ecological information for these species and all tropical reef fishes using rfishbase (Boettiger et al., 2012) and ggplot2 (Wickham, 2016). Finally, we examined the number of native, tropical, saltwater, reef‐associated fish species listed in rfishbase that are confirmed or likely to produce active sounds as listed on WoRMS (Boettiger et al., 2012; Looby et al., 2024; WoRMS Editorial Board, 2024).

## 3 RESULTS AND DISCUSSION

**FIGURE 1:**
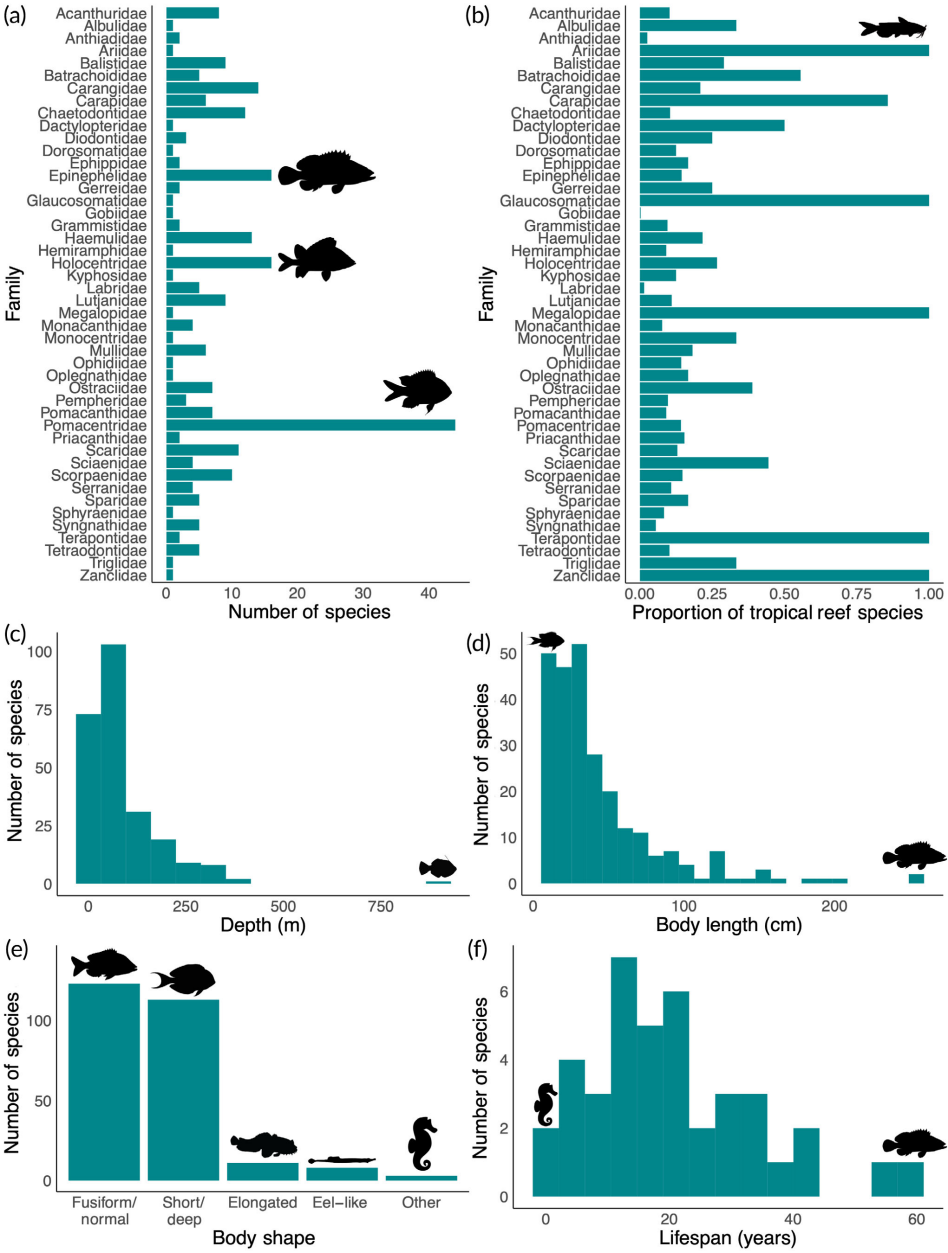
Taxonomy and characteristics of the 258 known actively soniferous tropical reef fish species, with representative images of select taxa (Schiettekatte et al., 2019). (a) Their abundance by family, (b) abundance relative to the number of tropical reef species within each family, and (c–f) prevalence across (c) maximum depths (bins = 15, *n* = 246), (d) body lengths (bins = 25, *n* = 256), (e) body shapes (*n* = 258), and (f) maximum lifespans (bins = 15, *n* = 40), as determined by FishBase (Froese & Pauly, 2023) and compiled from available data in rfishbase (Boettiger et al., 2012).

Active sound production was present in 258 tropical reef fish species, spanning 23 orders and 46 families. The families with the most soniferous species were Pomacentridae (damselfishes and clownfishes, *n* = 44), Epinephelidae (groupers, *n* = 16), and Holocentridae (squirrelfishes and soldierfishes, *n* = 16; Figure 1a). Species in these families have been studied relatively extensively for their sound production. For example, many clownfish species produce aggressive and submissive sounds important for their social hierarchies (Colleye & Parmentier, 2012), squirrelfish larvae are able to make sounds as soon as they settle on reefs (Parmentier et al., 2011), and grouper sound production has aided in monitoring their spawning aggregations (Malinowski et al., 2019). Many of the 46 families, however, still had a limited proportion of known soniferous species when considering the total number of tropical reef species in each family (Figure 1b). While five families were 100% soniferous, each of them only included one or two tropical reef species. For example, the hardhead sea catfish (*Ariopsis felis*), known for grunting choruses that sound like percolators (Fish & Mowbray, 1970), is the only tropical reef species in the family Ariidae. Nonetheless, 19 families were over 20% soniferous.

Soniferous tropical reef fish species were prevalent across a variety of depths, body lengths, body shapes, and lifespans. Most species studied so far occur in relatively shallow depths, with a median maximum depth of 50 m (Figure 1c). Several species occur deeper than 250 m (*n* = 15), with the orange filefish (*Aluterus schoepfii*), which produces sounds when competitive feeding and disturbed (Fish & Mowbray, 1970), occurring the deepest at 900 m. The body lengths of soniferous fishes ranged from the 6‐cm chocolate‐dip chromis (*Pycnochromis hanui*; Tricas & Boyle, 2014) to the 250‐cm Atlantic goliath grouper (*Epinephelus itajara*; Malinowski et al., 2019) and tarpon (*Megalops atlanticus*; Fish & Mowbray, 1970). The length data skewed towards smaller bodies, however (Figure 1d). All of FishBase's body shape categories were also represented, with, as expected, the two most common body shapes being fusiform (*n* = 123) followed by short and/or deep (*n* = 113; Figure 1e). While lifespan data were only available for 40 species, they nonetheless ranged in reported longevity in the wild from 1 year for the lined seahorse (*Hippocampus erectus*; Fish & Mowbray, 1970) to 60 years for the dusky grouper (*Epinephelus marginatus*; Desiderà et al., 2019; Figure 1f). The diverse characteristics of soniferous species demonstrate that sound production spans a variety of life‐history strategies and environments.

**FIGURE 2:**
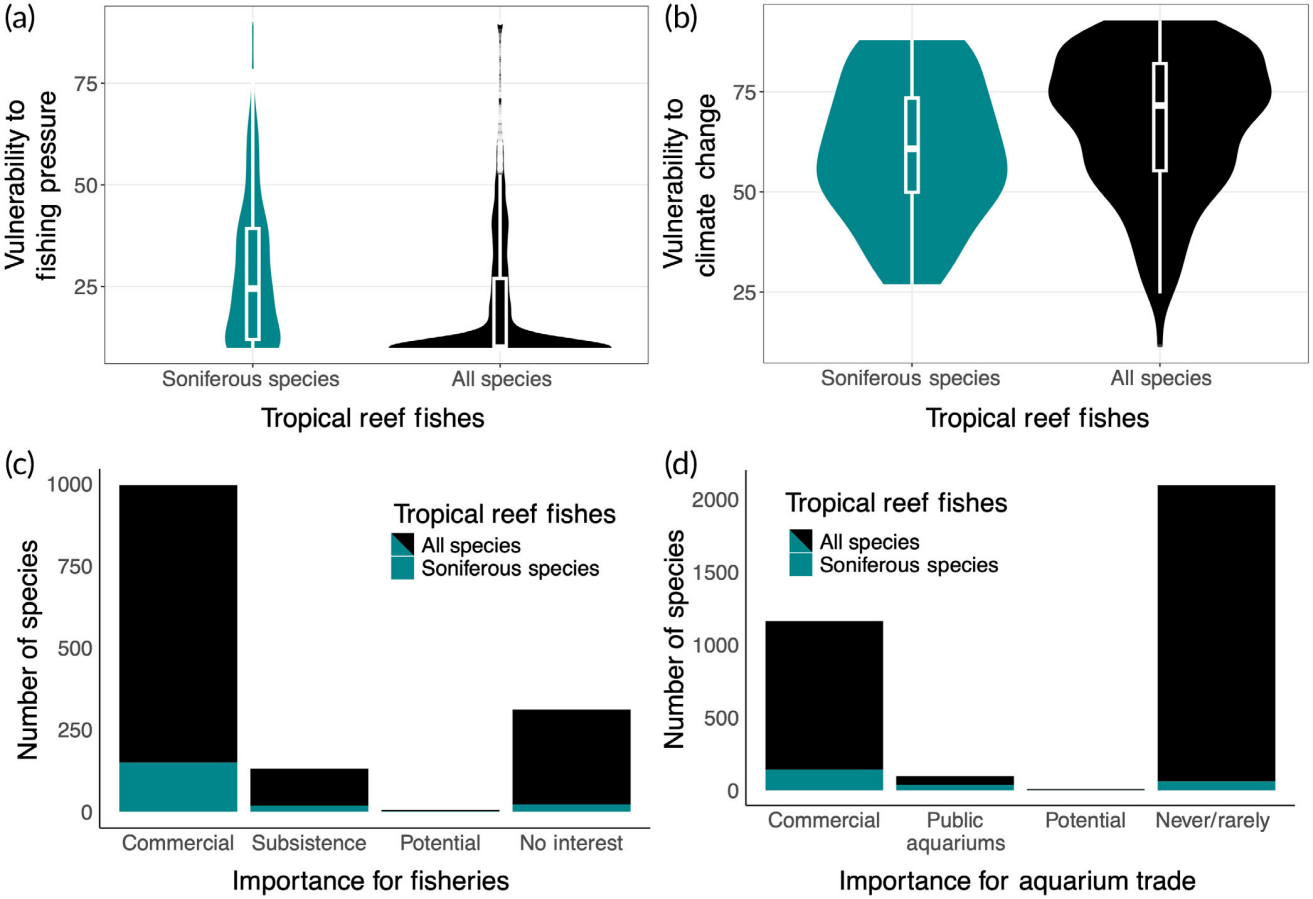
Vulnerability and commercial attributes of known actively soniferous and all tropical reef fish species. (a) Their vulnerability to fishing pressure (*n* = 258 and *n* = 3936) and (b) climate change (*n* = 34 and *n* = 156), with higher scores meaning species are more vulnerable to the associated pressure based on life‐history traits, as well as their (c) importance for fisheries (*n* = 191 and *n* = 1443) and (d) for aquarium trade (*n* = 239 and *n* = 3365), as determined by FishBase (Froese & Pauly, 2023) and compiled from available data in rfishbase (Boettiger et al., 2012).

Numerous soniferous tropical reef fishes are vulnerable and commercially valuable. FishBase provides indices of species vulnerability based on life‐history traits, with a higher score meaning species are more vulnerable primarily in the context of fishing pressure or climate change (Froese & Pauly, 2023). While most soniferous species had low vulnerability scores, four scored in the upper quartile for vulnerability to fishing pressure (Figure 2a), and of the 34 soniferous species assessed for vulnerability to climate, seven scored in the upper quartile (Figure 2b). These vulnerable soniferous species were mostly groupers, which tend to be relatively large, long‐lived, higher‐trophic predators and include species facing dramatic population declines caused by human activities (Froese & Pauly, 2023; Malinowski et al., 2019). In terms of value, 168 of the 258 soniferous tropical reef fish species are important for commercial or subsistence fisheries (Figure 2c) and 142 are used in the commercial aquarium trade (Figure 2d). Soniferous species were also overrepresented, with higher vulnerabilities to fishing pressure, commercial fisheries, and aquarium uses in comparison to all tropical reef fishes (Figure 2). Their commercial value may make these species at increased risk for population decline (e.g., Malinowski et al., 2019) and places an economic imperative for understanding whether their sound production behaviors can support monitoring, management, or conservation objectives (Myrberg & Fuiman, 2002; Duarte et al., 2021; Parmentier et al., 2021; Looby et al., 2022).

While we focused primarily on species reported to produce active sounds in the scientific literature, about 75% of tropical reef fish species are confirmed or considered likely to be actively soniferous based on an ancestral‐state reconstruction analysis and taxonomic relationships (Looby et al., 2024; Rice et al., 2022). This estimate could be considered an approximate upper limit for soniferous tropical reef species diversity based on current knowledge, although other studies support the expectation for active sound production to be widespread among tropical reef fishes. For example, field observations of Hawaiian coral reef fish communities recorded active sound production from 47% of 96 surveyed species, including during signal jumps of the Hawaiian damselfish (*Dascyllus albisella*), agonistic sounds of several triggerfishes (Balistidae), and pulse sounds produced by schooling bluestripe snapper (*Lutjanus kasmira*; Tricas & Boyle, 2014). Another soniferous diversity study of the coral reefs around Moorea Island (French Polynesia) found that 22% of the 241 fish genera present in the area contained known active sound producers and, if sonifery was extrapolated from the family level, as many as 67% of the genera could be soniferous (Parmentier et al., 2021). Although these estimates of sonifery range widely, they underscore the prevalence and importance of acoustic communication to a variety of fishes and their tropical reef ecosystems.

Our synthesis highlights the diversity, vulnerability, and value of a variety of soniferous tropical reef fishes, but our conclusions remain limited by the data available. Only about 4% of fish species globally have published documentations of sound production testing, likely biased towards species and sounds that are easier or more desirable to study as well as towards positive results of sound production testing (Bellwood et al., 2020; Fanelli, 2012; Looby et al., 2022). As a result, species not currently known to be soniferous could still be found to make active sound with further study and new soniferous fishes are being discovered every year (e.g., Fetterplace et al., 2022). Similarly, any assumptions of active sonifery based on taxonomy should be interpreted cautiously because of the independent evolution of sound production across fish taxa as well as the possibility for secondary loss (Looby et al., 2022; Rice et al., 2022). We were also limited to the taxonomic and ecological data available on rfishbase (Boettiger et al., 2012). Among the 258 species explored, 12 did not have available depth data, two did not have length data, 218 did not have lifespan data, 224 did not have vulnerability to climate change data, 67 did not have importance to fisheries data, and 19 did not have importance to aquarium trade data, with the additional possibility for errors in such an expansive database (Boettiger et al., 2012; Froese & Pauly, 2023). Even for attributes with limited sample sizes, the data were still able to showcase, for example, the wide range in soniferous fish lifespans (Figure 1f) and that at least some species are considered vulnerable to climate change (Figure 2b), even if their distributions may not be representative.

Current data limitations will be overcome with the continual growth in acoustic and diversity research, particularly if fish bioacoustics is treated as a scientific and management priority (Lindseth & Lobel, 2018; Looby et al., 2022; McKenna et al., 2021; Parmentier et al., 2021). Such growth could be spurred by emerging technologies in recording and analysis tools, systematic auditioning and reviews of tropical reef taxa and regions, global collaborations to improve data sharing, and public involvement in fish sound documentation (Lindseth & Lobel, 2018; Parmentier et al., 2021; Parsons et al., 2022; Tricas & Boyle, 2014). We hope this data synthesis and future efforts will help further elucidate the prevalence and importance of actively soniferous fishes to tropical reef environments globally.

## AUTHOR CONTRIBUTIONS

*Conceptualization*: Kieran Cox and Audrey Looby. *Methodology*: Emma Jayne Hodson, Kieran Cox, and Audrey Looby. *Formal analysis and investigation*: Emma Jayne Hodson and Audrey Looby. *Visualization*: Emma Jayne Hodson and Audrey Looby. *Writing – original draft preparation*: Emma Jayne Hodson. *Writing – review and editing*: Emma Jayne Hodson, Kieran Cox, Francis Juanes, and Audrey Looby. *Funding acquisition*: Emma Jayne Hodson and Francis Juanes. *Supervision*: Kieran Cox, Francis Juanes, and Audrey Looby.

## FUNDING INFORMATION

Emma Jayne Hodson was funded by a Mitacs Globalink Research Internship. The Liber Ero Foundation funded Kieran Cox and Francis Juanes. Audrey Looby was supported by a fellowship from the School of Forest, Fisheries, and Geomatic Sciences at the University of Florida.

## CONFLICT OF INTEREST STATEMENT

The authors have no competing interests to declare that are relevant to the content of this article.

## Supporting information


**Data S1.** The complete list of known actively soniferous tropical reef fishes and their associated taxonomic information and characteristics as collated from FishBase and FishSounds (Boettiger et al., 2012; Froese & Pauly, 2023; Looby et al., 2021, 2022).

## Data Availability

The data collated on known actively soniferous tropical reef fishes are included in the Supporting Information. All other data are publicly available from the FishSounds website data repository (10.5683/SP2/TACOUX), the WoRMS underwater sonifery data repository (10.5683/SP3/SVEXUS), and the R package, rfishbase (Boettiger et al., 2012).
